# Multisegment Extradural Tuberculoma Masquerading as a Spinal Tumor

**DOI:** 10.7759/cureus.24707

**Published:** 2022-05-03

**Authors:** Rishika Trivedi, Pankaj Trivedi, Rekha Gupta

**Affiliations:** 1 Medicine, Himalayan Institute of Medical Sciences, Dehradun, IND; 2 Neurosurgery, Vasal Hospital Neuro and Trauma Centre, Jalandhar, IND; 3 Pathology, Government Medical College, Amritsar, IND

**Keywords:** spinal tuberculosis, extradural, tuberculoma, laminectomy, spinal tumor

## Abstract

We report a case of a man who presented with features of spinal cord compression, and imaging showed an L3-S2 space-occupying lesion that mimicked a spinal tumor. The patient underwent L3 to S2 laminectomy and a fibrous, thick sheet-like, poorly vascular lesion was observed macroscopically. The histopathological examination of the lesion showed caseous necrosis and epithelioid giant cells. This raised suspicion of tuberculosis and which was later confirmed on a polymerase chain reaction. The present case illustrates that tuberculosis is a disease that should not be ignored, particularly considering its ability to resemble other types of mass lesions.

## Introduction

Tuberculosis (TB) is a chronic condition that has been demonstrated to damage nearly every organ of the body in a variety of ways. It has mimicked many disease pathologies, posing a diagnostic quandary. Extradural tuberculoma has been reported very infrequently. In most cases, inflammatory indicators such as C-reactive protein (CRP) are high in TB patients [1]. We report a case of a patient with spinal TB who required surgery but did not have an increased CRP; a multisegment space-occupying lesion on radiography led to the assumption that he had a spinal tumor. This is a rare case as the tuberculoma is extradural in location and spanned multiple spinal cord segments [2].

## Case presentation

**Figure 1:**
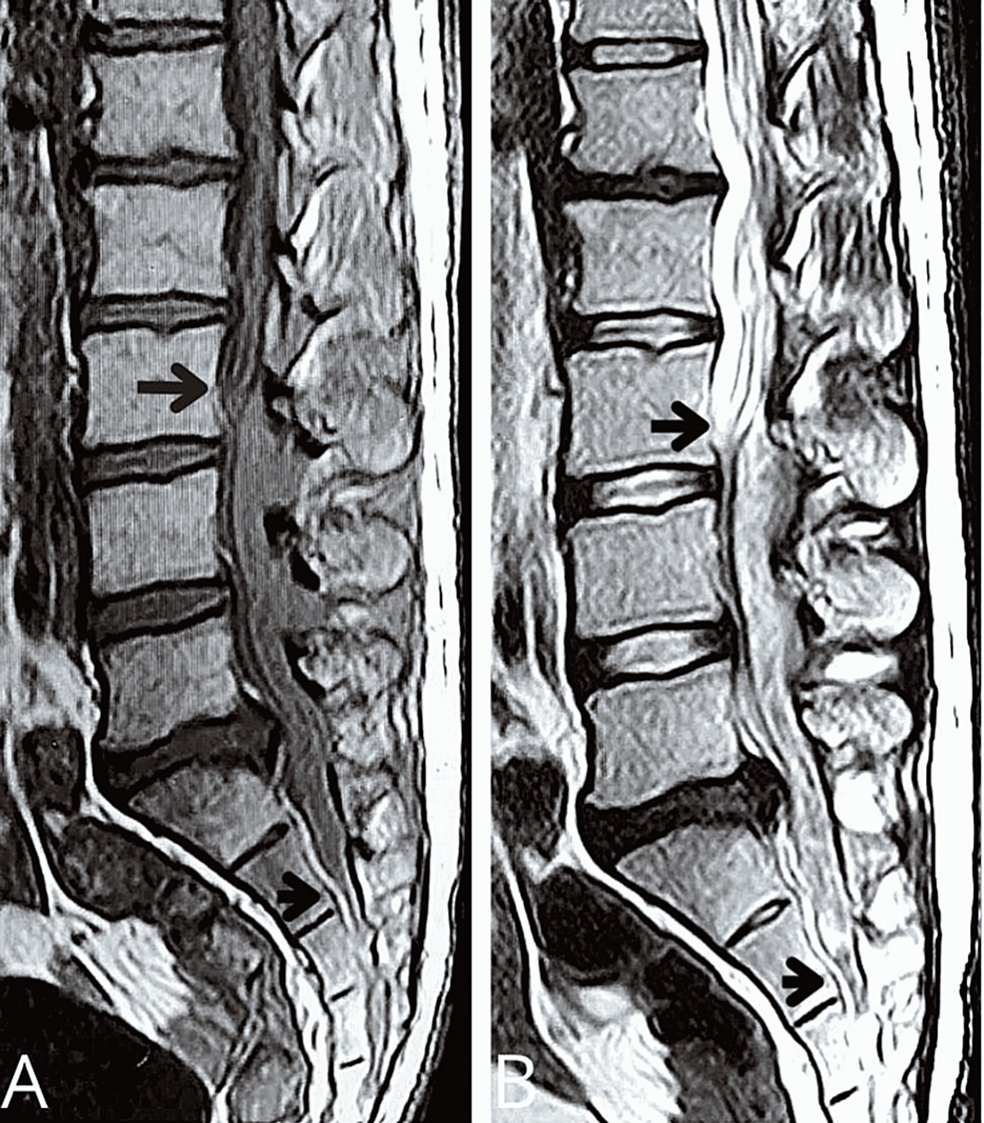
(A) Isointense lesion extending between L3-S2 segments in T1 MRI and (B) hyperintense lesion extending between L3-S2 segments in T2 MRI.

A 29-year-old man who was previously healthy came to the hospital Out-Patient Department with the chief complaints of difficulty in walking for the last two weeks, weakness, and foot drop bilaterally since two weeks. This was also accompanied by pain in the lower back and numbness in the thighs. There is no history of fever, trauma, focal weakness, and bladder and bowel involvement. There was no record of diabetes, hypertension, or TB. There is no history suggestive of contact with TB. Nutritional status and systemic examination are normal. The patient's neurological examination revealed bilateral lower extremity weakness and decreased knee reflexes bilaterally. There was no deformity noted on inspection. On MRI, there was a lesion (L3-S2) in the lumbosacral spine which was isointense on T1 MRI and hyperintense on T2 MRI (Figures 1A, 1B).

**Figure 2:**
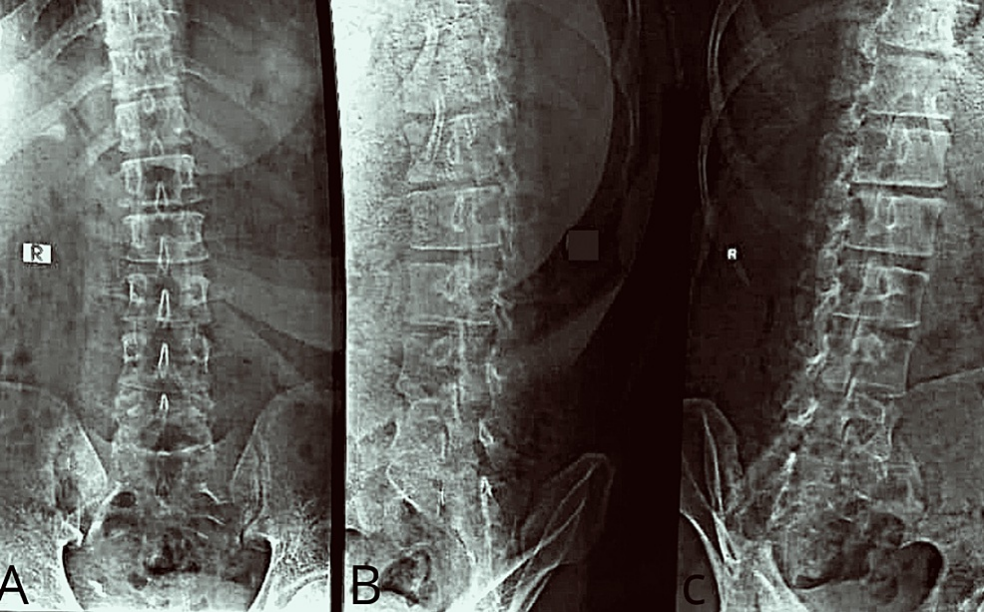
X-ray of the lumbo-sacral spine (A) antero-posterior view; (B, C) lateral view.

There were no fevers, and the laboratory data showed no elevation in the white blood cell count or CRP, the ESR measured 14mm/hour. There was no involvement of the vertebrae in the imaging, i.e., MRI and x-ray (Figures 2A-2C).

**Figure 3:**
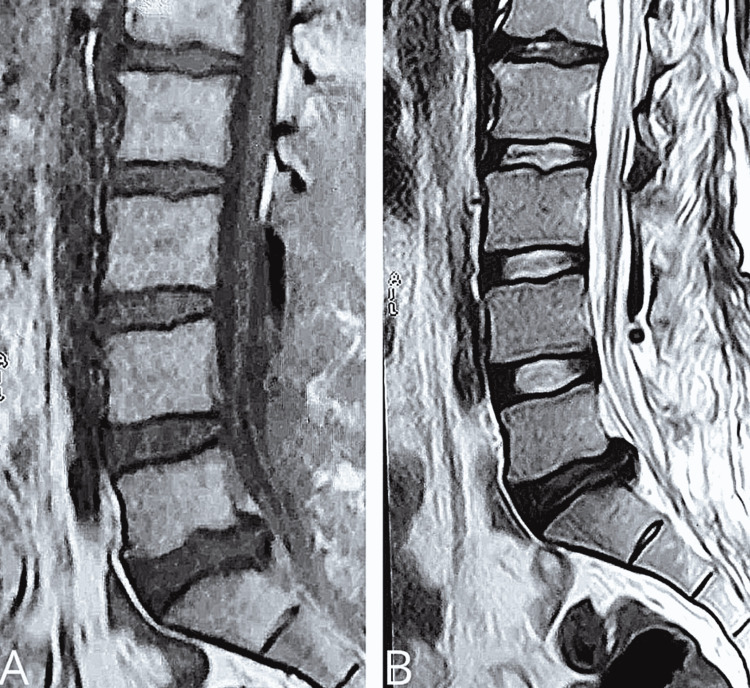
(A) T1 MRI and (B) T2 MRI images showing removal of the lesion.

The provisional diagnosis of benign neoplasm was considered after taking into account the clinical examination and imaging. The patient accepted to undergo surgical resection of the lesion due to the progressive neurological deficit. The patient went for L3 to S2 laminectomy with the removal of a fibrous and thick sheet-like poorly vascular lesion with liquefied yellow material near S1 and S2 levels. The dural sac became lax and pulsatile after decompression. The lesion spanned from the L3 to the S2 levels. Post-op MRI showed complete removal of the lesion (Figures 3A, 3B).

**Figure 4:**
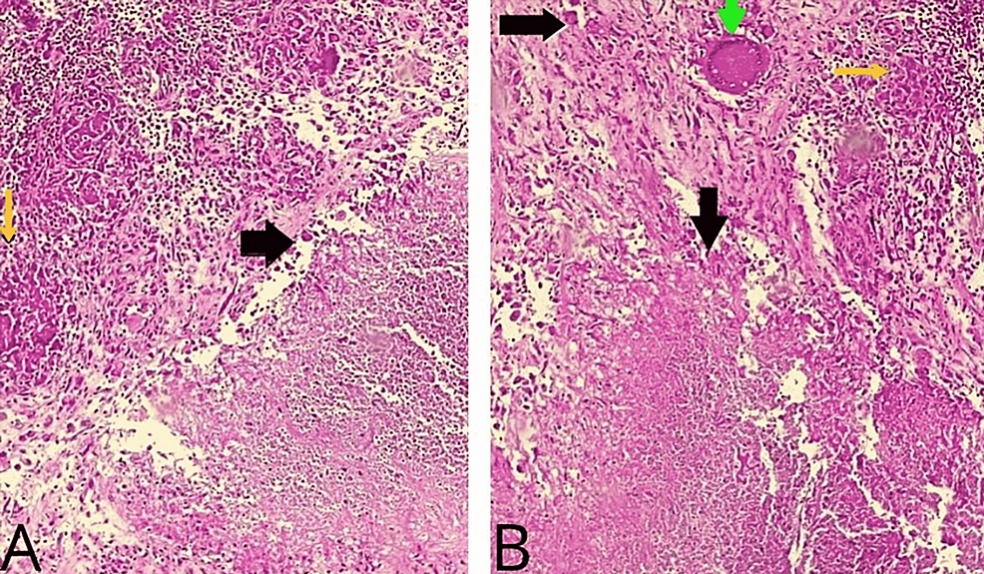
(A, B) Histopathological examination shows necrosis (black arrows) and granulomatous structures containing Langhans giant cells (green arrows) formed by epithelioid histiocytes (yellow arrows).

The necrotic material's histological analysis revealed In the histological analysis, histiocytes, lymphocytes, and necrotizing granuloma inflammatory reaction showed granuloma structures comprising Langhans giant cells generated by epithelioid histiocytes in fibro-adipose tissue with central necrotic foci (Figures 4A, 4B).

TB was suspected, and confirmation was made via a polymerase chain reaction. The chest x-ray revealed no abnormality. The neurologic function gradually improved after surgery and patient was started on an anti-TB treatment (ethambutol, rifampicin, and isoniazid) for nine months. At the six-month follow-up, the patient can walk independently without any discomfort.

## Discussion

TB accounts for an annual incidence of 2.0-2.3 million in the world, and India bears the highest burden of TB with about 150,000-350,000 deaths per year [3].

The clinical picture of TB varies; it is critical to increasing knowledge about the distinctive signs of the disease. One of the difficulties with TB is that the bacteria can remain dormant in many people for a long period before being reactivated to create the illness and its signs [4]. Spinal TB has no radiological characteristics in the early stages; simple radiography can only be useful after the bone has been destroyed. MRI is the gold standard since it aids in diagnosing, assessing the degree of the disease, and determining the surgical strategy [5]. The pathology finding is critical in determining the presence of spinal TB [6].

The most prevalent manifestation of TB is lung involvement, but CNS TB is more difficult to identify and has a poorer prognosis than pulmonary involvement. Because of the delay in therapy, recovery becomes even more difficult [7,8]. CNS TB is significant since it has a high death rate and affects 1%-5% of TB patients [9]. CNS TB can resemble a variety of different illnesses and creates a diagnostic difficulty owing to its presentation, which includes non-specific symptoms and inconclusive test results [10,11]. 

Neoplasms, primary central nervous system lymphoma (PCNSL), pyogenic abscess, fungal infection, cysticercosis, and toxoplasmosis should all be considered in the differential diagnosis of CNS tuberculoma [12].

Atypical spinal TB has the following characteristics: Involvement of the posterior elements of the spinal column with total sparing of the anterior elements (vertebral bodies and discs), skip lesions spaced by a sufficient distance, and extradural spinal cord compression with no radiographic indication of bone involvement [13]. The present case falls under the third category.

The granulomatous spine lesions that appear as spinal tumor syndrome are known as extradural tuberculoma. Because they exhibit symptoms of compressive myelopathy and no visible lesions on radiological examination, it has been proposed that they be classed as intraspinal tubercular granuloma [14]. Granulomatous foci form due to the hematogenous dissemination of mycobacteria to the central nervous system. Tuberculoma is a granulomatous lesion composed of epithelioid cells, Langhans giant cells, lymphocytes, and frequent central caseation. On macroscopic inspection, tuberculomas are spherical and encapsulated space-occupying lesions [9]. As we saw in this case, extradural TB can have an ambiguous presentation that is not always linked with constitutional symptoms, and in some situations, it can mimic a spinal tumor. Extradural tuberculoma, extradural extraosseous granuloma, intradural extramedullary TB, spinal arachnoiditis, and chronic adhesive arachnoiditis are all terms for granulomatous lesions of the spine that appear as spinal tumor syndrome. Because all of these appear as compressive myelopathy (spinal tumor syndrome) without evident radiological abnormalities, they have been classed as intraspinal tubercular granuloma [15]. Tuberculomas are typically up to 1cm in diameter, with around 10% being between 1 and 3cm and only rarely reaching 8cm [2]. The most common kind of TB paraplegia is vertebral TB with abscess, granulation tissue, or bone involvement. Our presents with spinal tuberculoma, which causes cord compression or cauda equina syndrome and has a history of extradural granulation tissue but no involvement of the bone. The patient experienced paraparesis as a consequence of extradural granuloma tissue pressure without bone involvement [16]. It is worth noting that there was no rise in CRP in our situation. Biomarkers such as WBC and CRP were often utilized to diagnose and assess therapy for pulmonary TB [1]. CRP rise is low in spinal TB, but no elevation of CRP is an incredibly unusual occurrence. As a result, we did not suspect spinal TB at first but rather pondered on spinal tumor. In fact, even with MRI or PET-CT, spinal TB is occasionally misdiagnosed as spinal neoplasm. However, pathology findings are also crucial in the diagnosis of spinal TB [6]. This case helps to consider that tuberculoma should be considered when dealing with mass lesions of the central nervous system, especially in the endemic regions.

## Conclusions

The diagnosis and treatment of extrapulmonary TB continue to be difficult for treating physicians. The differential diagnosis field is extensive, and symptoms of CNS TB frequently appear as unspecific in the early stages. Knowledge of radiological indications and potential mimics is expected to be useful. Although MRI may indicate distinctive neuroradiological signs of CNS TB, further clinical and laboratory evidence is required to make a diagnosis. This example serves as a reminder that TB is a disease that should not be overlooked, especially given its potential to resemble other mass lesions. Surgical decompression remains the backbone of therapy, with a satisfactory prognosis.
